# The role of central autonomic nervous system dysfunction in Takotsubo syndrome: a systematic review

**DOI:** 10.1007/s10286-021-00844-z

**Published:** 2022-01-08

**Authors:** Vojtech Brazdil, Petr Kala, Martin Hudec, Martin Poloczek, Jan Kanovsky, Roman Stipal, Petr Jerabek, Otakar Bocek, Martin Pail, Milan Brazdil

**Affiliations:** 1 Department of Internal Medicine and Cardiology, University Hospital Brno, Brno, Czech Republic; 2 Faculty of Medicine, Masaryk University, Brno, Czech Republic; 3 First Department of Neurology, St. Anne’s University Hospital, Brno, Czech Republic

**Keywords:** Takotsubo syndrome, Brain–heart axis, Insular cortex, Amygdala, Brain connectivity

## Abstract

**Introduction:**

Takotsubo syndrome (TTS), also known as stress cardiomyopathy or “broken heart” syndrome, is a mysterious condition that often mimics an acute myocardial infarction. Both are characterized by left ventricular systolic dysfunction. However, this dysfunction is reversible in the majority of TTS patients.

**Purpose:**

Recent studies surprisingly demonstrated that TTS, initially perceived as a benign condition, has a long-term prognosis akin to myocardial infarction. Therefore, the health consequences and societal impact of TTS are not trivial. The pathophysiological mechanisms of TTS are not yet completely understood. In the last decade, attention has been increasingly focused on the putative role of the central nervous system in the pathogenesis of TTS.

**Conclusion:**

In this review, we aim to summarize the state of the art in the field of the brain–heart axis, regional structural and functional brain abnormalities, and connectivity aberrancies in TTS.

## Introduction

**Fig. 1:**
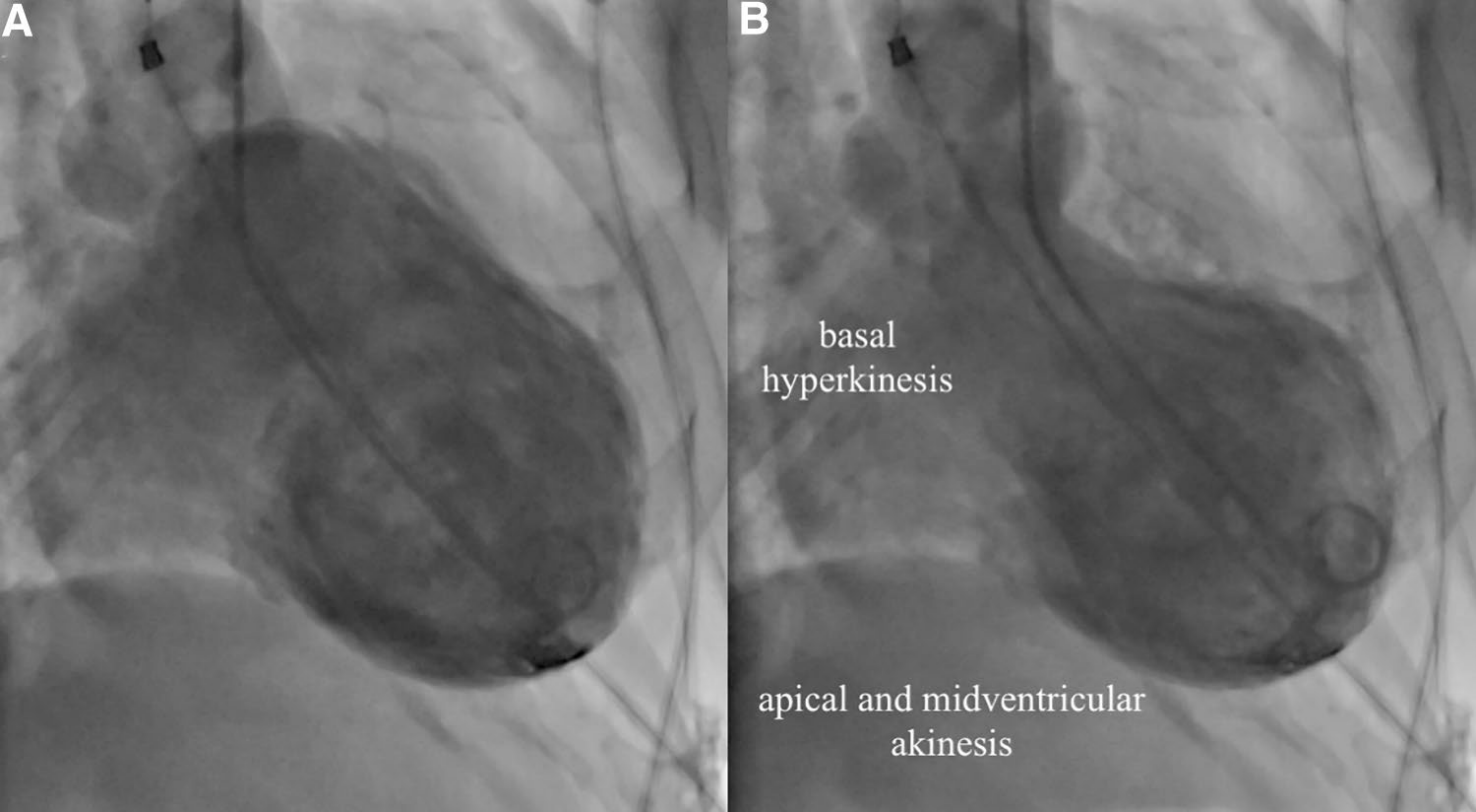
Left ventriculogram in acute TTS. **a** Diastole; **b** systole. Images demonstrate systolic dysfunction with hyperkinesis in the basal segments and akinesis of the apical and midventricular segments

Takotsubo syndrome (TTS) is an acute reversible left ventricular dysfunction that often mimics acute coronary syndrome (ACS). It may be challenging to distinguish TTS from ACS due to their similar symptoms, such as acute chest pain, dyspnea, and palpitations. In severe cases, TTS may manifest with ventricular arrhythmia or cardiogenic shock. Moreover, the diagnosis of TTS can be challenging because of the occurrence of electrocardiographic ( ECG) changes and elevated cardiac markers, which may also indicate the presence of ACS [1]. Approximately 1–2% of all patients treated for ACS are actually experiencing TTS. In the past, the prognosis for TTS was thought to be benign, but more recent studies have demonstrated higher short-term and long-term mortality than suggested from previous observations [2]. Mortality during the acute phase in hospitalized patients is currently estimated at 5% [3]. The primary diagnostic tool for TTS is coronary angiography and ventriculography (Fig. 1). Acute diagnostic coronary angiography reveals normal coronary arteries in most patients with TTS; however, about 15% of patients can have coincident coronary artery disease [4]. In these patients, the regional wall motion abnormality usually extends beyond the territory of the involved coronary artery [5]. Based on current findings, new diagnostic criteria were developed in 2018 (InterTAK Diagnostic Criteria) [1].

TTS is often triggered by a stressful event. Most cases occur in postmenopausal women with a mean age of 67–70 years (90%). The stressful triggers are sometimes, though not always, emotional, whether negative or positive. According to an analysis of the International Takotsubo Registry, the identified trigger is more often physical than emotional, and approximately one third of the patients have no evident trigger identified [4].

Several hypotheses concerning the pathogenesis of TTS have been proposed, such as microcirculatory dysfunction, transient ischemia induced by plaque rupture, multi-vessel epicardial spasms, and catecholamine toxicity on cardiomyocytes [1]. Although the exact pathophysiology of TTS is unclear, there is compelling evidence of enhanced central sympathetic stimulation. Stress reactions associated with the activation of the sympathetic nervous system and the excessive release of catecholamines seem to play a crucial role in the pathogenesis of TTS [6, 7]. Interestingly, TTS was repeatedly found to be triggered by several brain diseases, including subarachnoid hemorrhage, ischemic stroke, and epilepsy [8]. These diseases can be associated with excessive sympathetic stimulation as well [9]. In line with the analysis of the International Takotsubo Registry, more than half of the patients with TTS had an acute or chronic neurologic or psychiatric disorder [4]. These findings support the theory that the brain–heart axis plays a crucial role in the pathogenesis of TTS.

Recently, several studies have focused on the role of the central autonomic nervous system (ANS) in the pathophysiology of TTS. The ANS has a direct role in physical responses to stress and is divided into the sympathetic nervous system (SNS) and the parasympathetic nervous system (PNS).

This review article aims to provide an update of the rapidly evolving research on the role of the brain–heart axis in the pathophysiology of TTS and to discuss future directions for accurate early diagnosis and effective prevention of TTS.

## Methods

This systematic review was conducted and reported in accordance with the PRISMA statement for reporting systematic reviews.

### Literature search

A literature search of PubMed, Web of Science, and Scopus was conducted. The following search terms were used and combined: “takotsubo,” “central,” “nervous,” “brain,” and “autonomic.” A manual search was also performed using the reference lists of papers and conference abstracts to identify potential articles on the association between TTS and the central autonomic system.

### Inclusion and exclusion criteria

Criteria for the inclusion of publications in this systematic review were set a priori, as follows: (1) reporting of original data; (2) human studies of adults; (3) reported on patients diagnosed with TTS; and (4) written in English. Articles were excluded from our review if they were duplicate articles.

### Literature search results

**Fig. 2:**
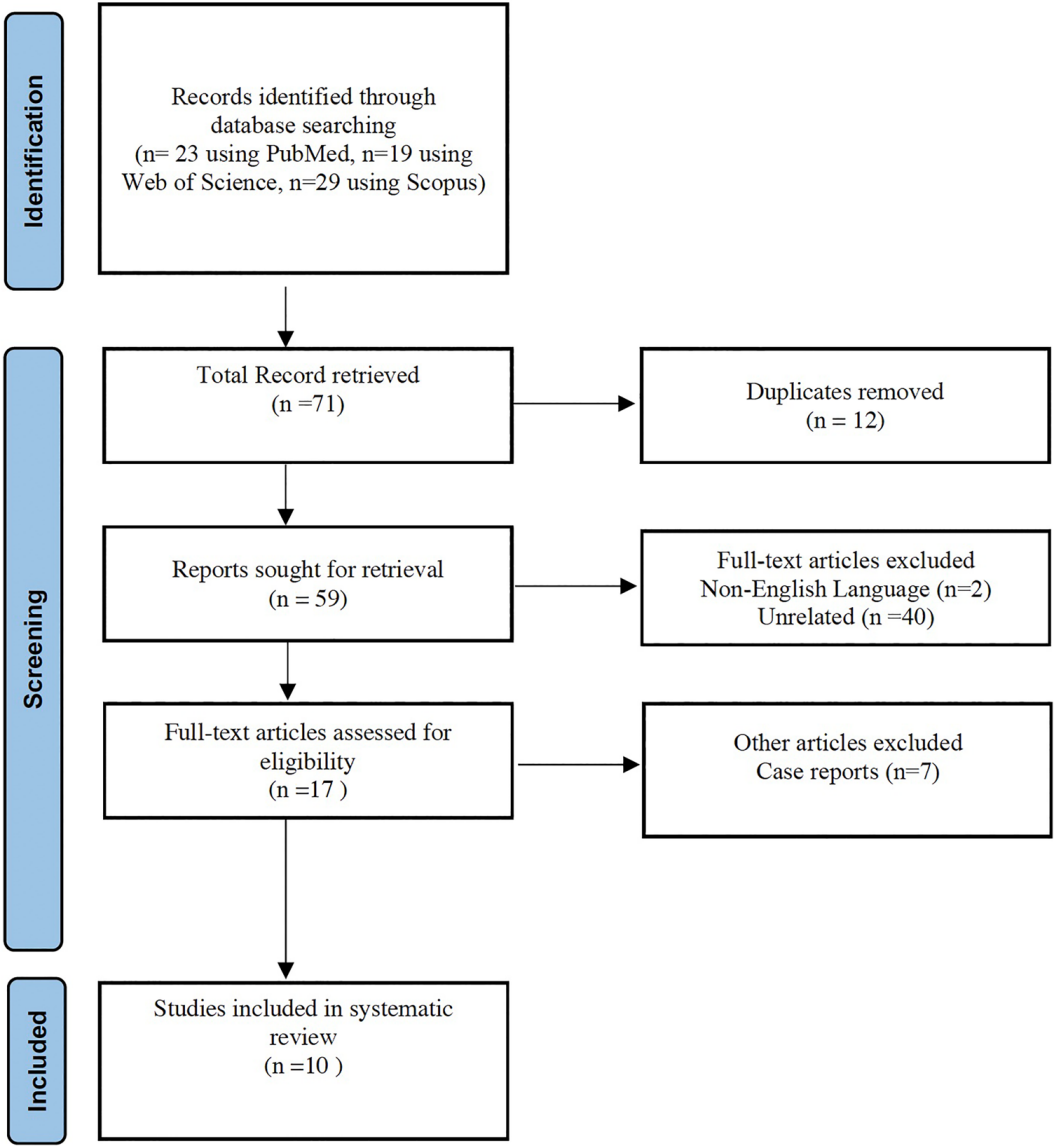
Flow diagram according to PRISMA guidelines for systematic review of the literature

A total of 71 articles were obtained. After evaluating the obtained articles, only 18 publications met the inclusion criteria for analysis. After excluding the duplicates, 10 articles remained. In total, 10 studies were included in this systematic review. Figure 2 represents a flow chart of the study selection. A meta-analysis was not appropriate because of the heterogeneity of the methodology and outcome assessments among the studies. Therefore, a narrative synthesis of the collected data was undertaken.

## Brain–heart axis

The connection between the brain and the heart was first noticed in the literature in 1846 by Sir George Burrows, who described the relationship between heart diseases and disorders of the cerebral circulation [10]. A few years later, Carl Ludwig announced the discovery of the depressor and accelerator nerves of the heart. In 1871, he and the American physiologist Henry Bowditch formulated the fundamental law of cardiac physiology: the “all-or-none” law of cardiac muscle [11]. However, it is only in the last few decades that we have begun to understand the physiology of the brain–heart axis. In the past, neurocardiological research focused on subcortical areas of the central ANS. Currently, it is generally accepted that the cardiovascular system is substantially regulated by the central autonomic network (CAN) consisting of three main nodes: the insular cortex, anterior cingulate cortex, and the amygdala. The CAN comprises regions in the brain that mediate the cardiac response to emotional and non-emotional stimuli by modulating the ANS and the hypothalamus–pituitary–adrenal axis [12]. These areas are involved in stress responses, emotional behavior, and homeostatic reflexes and exert their influence on the heart rate and cardiac contractility through the SNS and PNS.

## The insular cortex

One of the leading roles in CAN is played by the insula or, more specifically, by the insular cortex (IC). This brain region is highly interconnected with the cerebral cortex, basal nuclei, dorsal thalamus, amygdala, and other limbic areas. It has been suggested that the IC plays a prominent role in limbic-autonomic integration [13]. This mysterious brain structure, discovered by Johann Christian Reil in the 1800s, plays a role in many functions as the cortical region integrating emotions, memory, and autonomic and nociceptive activity. The literature has many case reports in which stimulation or damage of the IC resulted in various cardiovascular responses, including changes in blood pressure, QT dispersion, T wave inversion, and elevated troponin levels [14].

In 1990, Oppenheim et al. first described the direct link between the IC and cardiovascular regulation in an animal experiment. Using a new method of phasic insular microstimulation linked to the ECG cycle, they induced pure tachycardia independent of other autonomic or respiratory effects [15]. In another study 1 year later, they showed that prolonged phasic insular stimulation leads to an atrioventricular and interventricular heart block, QT interval prolongation, ventricular ectopy, and even death [16]. In 1992, the same group repeated a similar experiment in which stimulation was performed in five patients undergoing resective surgery for control of intractable epileptic seizures. This study demonstrated for the first time in humans that cardiovascular effects are obtainable by stimulation of the IC. This research revealed that while tachycardia was induced by stimulation of the rostral posterior insula, bradycardia was induced from the caudal posterior insula [16]. Some data suggested that there may be lateralization of cardiovascular control with sympathetic predomination of the right insula and parasympathetic cardiac neural regulation relating to the left insula [17]. Another study described the importance of IC in the management of the cardiac autonomic system by assessing the effects of acute right insular ischemic damage on heart rate variability and arrhythmia. The results of the study showed that more frequent heart rhythm disorders occur in a right-sided insular stroke [18].

Strokes involving the insular lobe are frequently associated with both bradycardia and heart blocks, as well as tachyarrhythmias with either atrial or ventricular origin [13, 19]. Focal seizures involving the insular lobe or amygdala, such as temporal lobe epilepsy, are also associated with autonomic dysfunction and cardiac changes [19]. Evidence from these studies indicates a crucial role of the insula in cerebrogenic cardiovascular disturbances. Research of the IC might provide critical insights into the nature of the pathophysiology of stress-related cardiovascular disease.

## Brain alterations in TTS

In recent years, several studies have been conducted using modern neuroimaging techniques, such as single-photon emission computed tomography (SPECT), positron emission tomography (PET), high-resolution structural magnetic resonance imaging (MRI), and functional magnetic resonance imaging (fMRI), to address the central regulation of the cardiovascular system in TTS [13, 20–26]. The focus has mostly been on specific brain regions involved in the cortical control of the autonomic system (IC, amygdala, cingulate cortex, hippocampus, and prefrontal cortex). The obtained data expanded knowledge about regional abnormalities of the brain at the structural and functional levels during the acute and chronic phases of TTS, and even before the disease itself. They also focused on brain connectivity at the structural and functional levels. Evidence from these studies suggests that functional alterations in the CAN are present in patients with TTS.

## 1. Regional abnormalities

(A) Perfusion

The very first direct evidence of brain involvement in TTS genesis was published by Suzuki et al. [20] in their SPECT study. The authors investigated changes in cerebral blood flow (CBF), which is a well-established index of brain activity. In three consecutive patients with TTS, they demonstrated a significant increase in CBF in the hippocampus, brainstem, and basal ganglia, and a significant CBF decrease in the prefrontal cortex within the acute phase of TTS. Not described in the paper but still clearly visible in Fig. 1 was a CBF increase within the right-sided IC. These changes in brain activity gradually subsided but were still present in the chronic phase (after recovery from the cardiac wall motion abnormality). It is worth noting that brain regions showing increased activity in this pioneering study are reported to be associated with sympathetic arousal, which is in line with a theory of excessive sympathetic stimulation in the development of TTS [20].

(B) Metabolism

The response to stressors such as those that could trigger TTS is governed by an ensemble of neural structures within the limbic system, which notably includes the amygdala [21]. A recent PET brain study in cancer patients presented very interesting results regarding this structure. Among 104 individuals with a high frequency of malignancy and with no structural brain lesions, 41 patients who subsequently developed TTS had a higher metabolic baseline ratio of amygdalar to regulatory (ventromedial prefrontal cortex) activity than those who did not. Moreover, the higher amygdalar activity was associated with a shorter interval between imaging and development of TTS. This heightened stress-associated neurobiological activity is present years before disease onset and may represent a previously unrecognized TTS risk factor [21]. Such heightened amygdalar activity may predispose individuals to TTS by potentiating the sympathetic, neurohormonal, and inflammatory consequences of future stressors [21]. Similar results were presented in a PET study by Tawakol. In this first longitudinal study to link regional brain activity to subsequent cardiovascular disease, heightened amygdalar activity independently and robustly predicted cardiovascular disease events. Amygdalar activity is involved partly via a path that includes arterial inflammation [22].

(C) Local brain volume

In a study published in 2018, Hiestand et al. focused on structural brain alterations and their role in the development of TTS. Changes in cortical thickness, surface area, and local volumes were evaluated using high-resolution structural MRI and subsequent surface-based morphometry (SBM) and voxel-based morphometry (VBM). Brain MRI scanning was performed in 20 female TTS patients 342 ± 274 days after the TTS episode, and 39 age- and gender-matched controls. SBM revealed significantly reduced cortical thickness in both-sided insulae and cingulate cortices in the TTS patient group. VBM analysis showed reduced gray-matter volume in the TTS subjects within three clusters: the left amygdala, right amygdala, and at the right amygdala/hippocampus border. The results demonstrated significant structural differences between the TTS patients and healthy control subjects in the limbic network comprising the insula, cingulate cortex, amygdala, and hippocampus [23]. However, the changes observed with such a long time interval may not be the cause but the consequence of TTS.

In 2020, an analogous study was completed by Dichtl et al. evaluating volumetric differences in gray matter using VBM in a cohort of 13 female TTS patients and 13 healthy controls. Here, MRI scanning was performed on the second or third day after hospital admission, and the acute TTS phase was investigated. In this case, structural volumetric changes cannot be expected as a consequence of TTS due to the presence at the time of TTS. TTS patients had significantly lower gray matter volume in numerous areas, particularly within the right middle frontal gyrus, which extended to the right insula, left central opercular cortex, right paracingulate gyrus, right and left thalamus, left amygdala, and right subcallosal cortex compared to controls [24].

In patients with TTS, reduced volume in some structures is common in both the acute and chronic phases of the disease, specifically in the right insula, the left amygdala, and the cingulate cortex. These regions are part of the CAN, which is involved in the regulation of the cardiovascular system.

(D) Functional alterations

Structural abnormalities and functional dysregulation within the central autonomic nervous system network were documented in TTS patients. In 2015, Pereira et al. published a fMRI study in which CAN responses to autonomic challenges (exposure to cold and Valsalva maneuver) were analyzed. The authors examined four patients with a previous episode of TTS and eight healthy controls. The results showed that some areas, specifically the IC, the amygdala, and the right hippocampus, respond abnormally to the Valsalva maneuver in TTS patients. During the Valsalva maneuver, there was an increase in the blood-oxygen-level-dependent (BOLD) signal in all of these regions, and significant differences in the activation patterns of indicated brain regions were proven between patients and controls [25]. It has been proposed that an impaired response to autonomic challenges and a dysregulation of the central autonomic nervous system network might contribute to the pathogenesis of TTS [25].

Another neuroimaging method that can be used to evaluate regional functional changes in the brain is resting-state functional magnetic resonance imaging (rs-fMRI). Resting-state fMRI allows researchers to explore the modular nature of cortical functions and to assess brain resting-state networks (RSN). The most fundamental RSN is the default mode network (DMN), which activates when a person is awake and not focused on the outside world. Sabisz et al. studied the critical areas of the DMN, which are located in the posterior cingulate cortex/precuneus and medial prefrontal cortex. Using rs-fMRI, the authors found increased activity in both-sided precuneus and decreased connectivity in the ventromedial prefrontal cortex in 13 TTS subjects as compared to healthy controls [26]. Regarding the reported enhanced anxiety levels and the often experienced negative effects of the TTS patients, Sabiz et al. suggested that this increased focus on internal and self-regulation processes might reflect an inefficient emotion regulation mechanism in these patients.

## 2. Connectivity abnormalities

(A) Structural connectivity

Possible brain alterations in TTS patients can be demonstrated by changes in structural connectivity when using diffusion tensor imaging (DTI) data. Hiestand et al. focused on differences in connections among brain regions controlling the ANS. Their analysis of structural brain connectivity at the level of the whole brain network showed reduced connectivity, mainly within the limbic system, in 20 TTS patients. While focusing on connectivity within the ANS, the TTS patients showed a reduction of fibers among both hippocampi, left amygdala, left parahippocampal gyrus, left superior temporal pole, and right putamen. The results of this study thus clearly demonstrated significant differences in structural connectivity in the limbic network, which is strongly involved in the control of emotional processing, cognition, and the ANS [23].

However, these findings were not confirmed in a smaller cohort of TTS patients investigated by Sabisz et al. These authors computed fractional anisotropy (FA) as a DTI method in 13 TTS patients and compared the data with a healthy cohort. No significant differences in FA values were revealed [26].

(B) Functional connectivity

Using rs-fMRI, Templin et al. studied changes in functional connectivity patterns in TTS patients at the level of particular ANS subnetworks (SNS and PNS), the DMN, and at the whole-brain level. When compared to healthy controls, 15 TTS patients revealed, in the chronic phase of the disease, reduced resting-state functional connectivity in the SNS and the PNS, comprising mainly the amygdala, hippocampus, and IC, but also the cingulate, parietal, temporal, and cerebellar regions. The analysis of the DMN and the whole-brain network showed reduced functional connectivity in specific areas of the limbic/autonomic nervous system, specifically in the hippocampus, and the parahippocampal and medial prefrontal regions. Not surprisingly, reduced functional connectivity has been observed in TTS patients in similar areas of the brain, where structural changes were present in previous studies [27].

In parallel, Sabisz et al. investigated functional connectivity using rs-fMRI and studied the critical areas of the DMN located in the posterior cingulate cortex/precuneus and medial prefrontal cortex. In their study, the DMN component in TTS patients showed increased connectivity areas in the precuneus. The TTS patients had decreased connectivity in the ventromedial prefrontal cortex, as compared to the healthy controls [26].

Brain functional connectivity was further investigated in a study by Silva et al. in 2019. Here, the authors focused on changes in eight TTS patients both at rest and during stressful stimulation (cold exposure). In the resting state, they observed increased connectivity in a network composed of the left anterior insula, left anterior cingulate, superior temporal cortices, left inferior frontal cortex, left hippocampus, and left parahippocampal cortex. The authors observed increased functional connectivity in a network composed of nodes located in the amygdala, left putamen, right insula, and right cerebellum, among others, in the TTS patients after exposure to a stressful stimulus, as compared to TTS patients at rest and healthy controls. As described above, some of these areas are crucial parts of autonomic and emotional control [28].

Very recently, Dichtl et al. were the first to describe changes in functional connectivity in TTS patients in the acute phase of the disease. Using graph analysis, lower functional connectivity was observed in patients, particularly in the connections from the right anterior IC, temporal lobes, and right precuneus, compared with controls. In the same study, the authors found that specifically the right insula, associated with the sympathetic autonomic tone, had both volumetric and functional changes [24].

## 3. A predictive model based on brain imaging data

An extremely challenging study was recently performed by Klein et al., who did a multivariate pattern analysis using machine learning with multimodal MRI data of the human brain of 20 TTS patients. In this breakthrough study, they aimed to identify predictors for the presence of TTS based on MRI data, with the most promising being DTI and rs-fMRI. They demonstrated homogeneous structural and functional neuronal alterations in TTS patients and utilized anatomical and neurophysiological measures from brain regions constituting the emotional-autonomic control system for disease prediction with an accuracy of more than 82% [29]. Interestingly, this study revealed alterations in several of the brain areas that form the “central part” of the autonomic nervous system controlling cardiovascular functions via both the SNS and PNS. Furthermore, given the fact that the authors identified brain regions to be predictive for TTS that are involved in the control of both nervous systems, this may suggest that an impaired mutual interference of both these nervous systems could also be an underlying cause of TTS [29].

## Discussion

The involvement of the brain–heart axis has been proposed in TTS. Primarily, excessive activation of the SNS has been suggested as playing a crucial role in the development of TTS. These findings were strongly supported by the research by Akashi et al., who first demonstrated an increased sympathetic activity and excessive catecholamine stimulation of the myocardium using ^123^I-metaiodobenzylguanidine (MIBG) myocardial scintigraphy. MIBG is an analog of norepinephrine, which can be used to assess the density of sympathetic myocardial innervation. The role of sympathetic hyperactivity was further confirmed by direct measurements of sympathetic nerve activity [30]. Vaccaro et al. used the evaluation of spontaneous baroreflex control of sympathetic activity and microneurography, a powerful method to evaluate the sympathetic tone. In their study, the authors directly demonstrated increased SNS activity associated with decreased spontaneous baroreflex control of sympathetic activity in patients with TTS [31]. It is well known that abnormalities in wall motion usually extend beyond a single epicardial coronary artery; now it appears that the wall motion patterns in TTS are associated with sympathetic innervation of the heart and correlate with the distribution of the myocardial sympathetic nerve terminals [32]. While the role of the sympathetic nervous system is heavily discussed, the role of PNS has often been neglected. But Norcliffe-Kaufmann et al. assessed sympathetic and parasympathetic activity using baroreflex, cognitive, and emotional stimulation; the results revealed a decrease in parasympathetic modulation of heart rate in TTS patients [33]. The role of CAN has also been heavily studied recently in parallel; this is also the main focus of our review.

Recent evidence from neuroimaging studies has strongly suggested that alterations in the CAN are present in patients with TTS. Studies based on limited numbers of patients have repeatedly demonstrated both structural and functional alterations at the regional level as well as significant changes in brain connectivity. Publications focused on regional abnormalities studied changes in perfusion [20], metabolism [21, 22], local brain volume [13, 23], and functional properties [25, 26]. These studies demonstrated the presence of altered neural networks in several stress-associated limbic brain regions (i.e., the amygdala, insula, anterior cingulate cortex, prefrontal cortex, and hippocampus) in patients with TTS in both the chronic and acute phases of the disease, and even before the development of TTS. Even if the results from numerous neuroimaging studies of TTS can hardly be questioned, they do not directly touch on the regulatory influence of the hypothalamic and brainstem nuclei. Indeed, structural lesions within the rostroventrolateral medulla, solitary tract nuclei, and other brainstem structures have also been repeatedly linked to the development of TTS [34–36]. Importantly, these parts of the CNS play a vital role in the regulation of cardiac functions as well, and thus their participation in the pathogenesis of TTS can be presumed, possibly as the important nodes of the relevant neural networks.

Abnormalities in brain connectivity were studied at two levels: changes in structural connectivity [23, 26] and changes in functional connectivity [24, 26–28]. Practically all these studies congruently revealed changes in both connectivity types in patients with TTS. Studies have shown that individuals with prior TTS manifest abnormal neural connectivity among regions associated with autonomic function and limbic system regulation [37].

Importantly, information based on alterations in anatomical and neurophysiological measures from brain regions involved in the emotional-autonomic control system can serve as possible predictors of TTS with an accuracy of more than 82% [29]. The results of the given studies intersect in numerous points and indicate the most common occurrence of alterations in the insula, cingulate, amygdala, and hippocampus. These fundamental areas of the limbic/autonomic system control emotions, learning, or memory. Heightened activity within such stress-associated neural tissues augments SNS and has recently been shown to contribute to the pathogenesis of multiple chronic stress-related diseases [3, 22]. This construct is also consistent with observations of decreased (ventromedial) prefrontal cortex perfusion and metabolism, and altered connectivity among individuals with prior TTS and with subsequent TTS [20, 21, 22, 26], given the important role of the prefrontal cortex in reducing the stress response.

The amygdala is strongly involved in cardiac control and is one of the main structures of the sympathetic nervous system that controls the nervous system’s response to stress. It also plays a major role in the perception of fear, mental and post-traumatic stress, depression, and anxiety disorders [29]. The amygdala is not only associated with negative emotions; it has recently been implicated in the processing of pleasant emotions such as happiness. TTS occurs in 27% of patients in relation to emotional stress factors. TTS is typically provoked by negative stressors; however, there are references to positive emotional TTS triggers in 1% of cases. Nagai et al. described an episode of TTS triggered by positive emotion in a patient with left internal carotid artery (ICA) occlusion. In this case, TTS may be associated with ICA occlusion and IC hypoactivation [38]. Some studies have suggested that the left IC is associated with the parasympathetic autonomic tone. The authors hypothesized that ICA occlusion may have led to decreased PNS and impaired sympathovagal activity with a predominance of the SNS. They further stated that a positive stress factor could lead to increased SNS activity in the right hemisphere. These findings led to the idea that whether there is a positive or negative trigger at the beginning, there is a common pathway of processing and CNS output in the origin of TTS [38].

The hippocampus is a core component of the limbic system, which is responsible for the processing of emotional stimuli and memory formation and also has a role in central autonomic modulation [28]. It is mainly the IC that has a pivotal role in the integration of autonomic, motor, and sensory functions through its reciprocal connections with other parts of the brain, notably the amygdala, the anterior cingulate cortex, and the hippocampus. This area is considered to be primarily responsible for integrating emotional, cognitive, and social stimuli in the autonomic response. Furthermore, this region is thought to be involved in the afferent and efferent pathways of the autonomic system, indicating the IC as the main regulator of the cortical autonomic responses. It has been repeatedly observed that focal IC pathology, such as limited insular strokes or epileptic seizures originating from the insular cortex, could be associated with arrhythmias and other autonomic dysfunctional manifestations including TTS [28, 39].

All these findings suggest that dysregulation of the CAN may play an important role in the pathophysiology of TTS and underscore an essential role of the brain–heart axis in TTS. On the other hand, despite the convincing occurrence of brain alterations in TTS patients, it cannot be ruled out that these functional and anatomical changes are a consequence rather than a cause of cardiac dysfunction. However, evidence from recent studies points more to autonomic dysfunction as the cause of cardiac dysfunction. This hypothesis is substantiated both by the observed structural changes in the stress-associated neural centers in the limbic system that are evident shortly after the onset of TTS [24] and by the functional changes observed even before the onset of TTS [21, 22]. Based on the data, this neural network plays a critical role in responding to stressors via its efferents, affects physiologic changes through the SNS, and thus may initiate complex pathophysiologic cascades [1]. Noradrenergic neurons in the brainstem and the sympatho-adrenomedullary axis are activated, causing the release of catecholamines, and likely contributing to TTS [3]. It is conceivable that TTS is triggered by neuronal alterations in the limbic system, presuming an emotional hypersensitivity or disturbed emotional processing in these patients. In accordance with this statement, Templin et al. showed that rates of neurologic or psychiatric disorders were higher in a substantial number of TTS patients (55.8%) compared to ACS patients (25.7%) [3]. To summarize, the presented studies shed light on a “heart–brain connection” representing a neurobiological mechanism that contributes to TTS. To confirm these hypotheses, future research, especially prospective research, in this field is therefore extremely welcome.

## Conclusions

Recent neuroimaging studies of TTS have provided relatively strong evidence for the presence of structural and functional alterations in stress-associated neural centers in the limbic system. Although these findings have elucidated the possible involvement of the brain–heart axis in the pathogenesis of TTS, many questions remain. Can we identify patients with classical stress cardiomyopathy who are at high risk? Are CAN alterations present in all patients or in “neurologic” patients only? Are we observing a cause or a consequence, or both? And finally, if classical TTS is indeed a neurocardiological disease, what is the underlying pathophysiological mechanism of the central dysfunctions? To accurately understand the role of the limbic and autonomic nervous systems in the development of TTS, it is necessary to perform prospective and very well-controlled longitudinal studies with larger samples of patients.

Further research in this area very likely could help expand diagnostic and therapeutic options. In parallel to current diagnostic approaches and promising avenues, including the detection of circulating microRNAs for accurate identification of TTS in the acute phase, neuroimaging techniques might provide an additional tool for improving the diagnosis. Assuming a CAN alteration as the cause of TTS development, our point of interest could shift from the heart to affecting primarily the brain and possibly both pharmacologic and non-pharmacologic behavioral interventions, such as stress reduction, for preventing the disease.
